# Age-dependent oral manifestations of neurofibromatosis type 1: a case–control study

**DOI:** 10.1186/s13023-022-02223-x

**Published:** 2022-03-02

**Authors:** Eshwar Thota, John Jims Veeravalli, Sai Krishna Manchala, Bhargavi Priya Lakkepuram, Jayasurya Kodapaneni, Yi-Wen Chen, Li-Tzu Wang, Kevin Sheng-Kai Ma

**Affiliations:** 1 Panineeya Institute of Dental Sciences and Research Centre, Hyderabad, Telangana India; 2 SVS Institute of Dental Sciences, Mahbubnagar, Telangana India; 3 Department of Dentistry, National Taiwan University Hospital, Taipei, Taiwan, ROC; 4 Graduate Institute of Clinical Dentistry, School of Dentistry, National Taiwan University, Taipei, Taiwan, ROC; 5 Institute of Cellular and System Medicine, National Health Research Institutes, Zhunan, Taiwan, ROC; 6 Graduate Institute of Biomedical Electronics and Bioinformatics, College of Electrical Engineering and Computer Science, National Taiwan University, Taipei, Taiwan, ROC; 7 Center for Global Health, Perelman School of Medicine, University of Pennsylvania, Philadelphia, PA USA

**Keywords:** Neurofibromatosis type 1, Oral complications, Periodontitis, Salivary gland dysfunction, Dental caries

## Abstract

**Introduction:**

Most craniofacial manifestations of neurofibromatosis type 1 (NF1) are considered as a result of tumor compression. We sought to determine salivary changes, caries, and periodontal complications in NF1 patients without tumors in the oral cavity.

**Objective and methods:**

Eleven NF1 patients without tumors in the oral cavity and 29 matched controls without NF1 were enrolled in this case–control study. Demographic information, medical history, and data of intraoral examinations, including the Decayed, Missing, and Filled Teeth (DMFT) scores and Russel’s periodontal index (PI), were recorded. The functional salivary analysis was performed for sialometry, salivary pH values, and amylase activity. Ingenuity Systems Pathway Analysis (IPA) was conducted to identify mutually activated pathways for NF1-associated oral complications.

**Results:**

NF1 patients were associated with periodontitis (OR = 1.40, 95% CI = 1.06–1.73, *P* = 0.04), gingivitis (OR = 1.55, 95% CI = 1.09–2.01, *P* = 0.0002), and decreased salivary flow rates (OR = 1.40, 95% CI = 1.05–1.76, *P* = 0.005). Periodontal destruction, salivary changes, and dental caries in NF1 patients were age-dependent. Subgroup analyses based on age stratification suggested that salivary flow rates and salivary amylase activities were significantly low in NF1 patients aged over 20 years and that salivary pH values, PI and DMFT scores were significantly high among NF1- controls aged over 20. All oral complications were not significantly presented in NF1 patients aged below 20 years. IPA analyses suggested that cellular mechanisms underlying NF1-associated oral complications involved chronic inflammatory pathways and fibrosis signaling pathway.

**Conclusion:**

NF1 patients without tumors in the oral cavity presented a comparatively high prevalence of age-dependent oral complications, including periodontal destruction and salivary gland dysfunction, which were associated with chronic inflammatory pathogenesis.

## Introduction

Neurofibromatosis is a complex group of syndromes which occurs due to the inactivation of the various tumour suppressor genes precipitating various complications in the body [1, 2]. Classified into eight types, the most common type of neurofibromatosis that accounts for 90% of all cases is neurofibromatosis type 1 (NF1), which is underlain by germline mutations in the NF1 gene (locus 17q11.2) [1]. NF1 is inherited in an autosomal dominant pattern, with a penetrance of 100% by age of 20 years [1]. In 50% of cases, the mutated gene is inherited from the parents, and in the other 50% of cases, mutations develop spontaneously [1]. The mutated genes expressing rapidly in females [1].

The NF1 gene is ubiquitously expressed in all cells of the body, but the phenotypic expression differs from cell to cell and within a cell through different developmental stages of life [1, 2]. Hence, apart from tumours involving the nervous system, NF1 is associated with cutaneous angiomas, subcuta neous leiomyomas, carcinoid tumours, and pheochromocytoma, as well as auditory, visionary, cognitive, sleep [3], musculoskeletal, endocrinal and cardiovascular complications [4]. Endocrinological, neurological, and ophthalmological complications of NF1 are age-dependent, as driven by hormone imbalance at puberty or during pregnancy, and usually  are accompanied with the progression of neurofibromas in terms of their numbers and sizes [5]. Severe complications of NF1 also include the risk of malignant peripheral nerve sheath tumours (MPNSTs) [1].

Due to its rarity, there has been a lack of studies reporting the oral complications of NF1, with findings of these works including an asymmetrical enlarged face with multiple cutaneous/disseminated and plexiform neurofibromas [1, 2, 8–10, 13, 14]. In addition to sphenoid and orbital bone dysplasia, mandibular bony changes such as deformities of the mandibular ramus and glenoid fossa (56%), condylar head (50%), increased dimensions of the coronoid notch, decreased jaw angle, and enlarged mandibular canals (25%) have been noted [1, 2, 8–10, 13, 14]. These changes are accompanied by facial plexiform neurofibromas along the trigeminal nerve that can cause cephalometric alterations. Other common intraoral findings include pigmentation of oral mucosa [6, 7, 15] and tongue, enlarged tongue and fungiform papillae, malposed teeth with an increased incidence of dental caries, periapical cemental dysplasia, perineural fibrous thickening of pulpal tissue and class III malocclusion [8–10]. Given that periodontal disease may take place following reported craniofacial complications of NF1 such as malocclusion and hyposalivation [11], and that functional salivary changes were yet to be determined in previous studies [12, 13], we conducted this study to identify the above-mentioned orofacial complications in NF1 patients, and evaluated whether these symptoms were, similar to that observed within endocrinological complications of NF1 [5], age-dependent [1].

## Materials and methods

### Study design

In this case–control study, 11 NF1 patients of NF1+ status that met the diagnostic criteria for NF1 by the National Institutes of Health [1, 12, 13], and 29 matched controls without NF1 (NF1-), were recruited at Panineeya institute of dental sciences and research centre, India. The diagnosis of NF1 required at least two of the following criteria: (1) Six or more café-au-lait spots or hyperpigmented macules greater than 5 mm in diameter in prepubertal children and greater than 15 mm post-pubertal, (2) axillary or inguinal freckles, (3) at least two typical neurofibromas or one plexiform neurofibroma, (4) optic nerve glioma, (5) at least two iris hamartomas or Lisch nodules in slit-lamp examinations, (6) sphenoid dysplasia or typical long-bone abnormalities. The 29 NF1- controls were matched on their age, gender, residing region, socioeconomic status, personal and deleterious habits, and history of medications [16–18] which can influence our primary outcome, the salivary flow rate. To ensure the observed oral complications did not result from direct tumour compression, it was required in this study that all enrolled NF1 patients did not have tumors in the maxilla or mandible. None of the NF1 patients had a diagnosis of Legius syndrome. This study follows the Strengthening the Reporting of Observational Studies in Epidemiology (STROBE) Statement observational studies, the Declaration of Helsinki, and the National Statements of Ethical Conduct Elements. Informed consent was acquired from all participants and was approved by the ethical committee.

### Data and intraoral findings

Demographic information and medical history were collected. After conglomeration of data, clinical examination was performed using a mouth mirror and William’s periodontal probe. For the intraoral findings, the Decayed, Missing, and Filled Teeth (DMFT) index and Russel’s periodontal index (PI) values were recorded. Accordingly, the diagnoses of gingivitis and periodontitis [19–23] were made based on Russel’s periodontal index score. RPI scores of 0–0.2, 0.3–0.9, 1–1.9, 2–4.9, 5–8 were classified as clinically normal supportive tissues, simple gingivitis, beginning of the destructive periodontal disease, established destructive periodontal disease, terminal stage of periodontal disease, respectively [24].

### Collection of saliva for salivary analysis

To evaluate their salivary flow rates, sialometry using whole unstimulated saliva was performed in the morning between 10 to 11 AM. Patients were instructed not to brush or use alcohol/ non-alcohol-based mouthwashes, consume food or liquids 3 hours before the examination. The individuals were asked to rinse their mouths with water and after waiting 10 minutes, the saliva was collected in a sterile test tube for one minute [13, 25]. Accordingly, the salivary flow rate was defined as normal when the whole unstimulated salivary flow rate was greater than 0.3 milliliter per minute (ml/min), decreased salivary rate if the values were in between 0.3 and 0.1 ml/min, and hyposalivation if the values were less than 0.1 ml/min [13].

To identify the salivary pH values and salivary amylase activity (1/min), 1 ml of whole unstimulated saliva was collected in a sterile test tube and the pH value of saliva was measured using a digital pH meter (Electronics India, Model 101). The entire solution was made to 50 ml by adding sterile water. To this solution, 5 drops of 1% starch solution (QUALI-TECH CHEM Starch Indicator 1% Solution 99% PURE) and 2 drops of iodine reagent (Bio balance Lugol’s 2%) were added along the walls of the test tube. After mixing thoroughly, the colour change of the solution into the blue was set as the index timepoint, and from that point, the time taken for the solution to become transparent was recorded [26].

### Statistical analysis

The comparison of the NF1 patients and matched controls (NF1-) for each age subgroup was done by using *t* tests. *P*-values less than 0.05 were considered to be significant. Age distribution of sicca syndrome and periodontitis among NF1 patients and NF1- controls were demonstrated, and the chi-square test was used to test the significance. The odds ratios (ORs) were derived to estimate the risk of sicca syndrome and periodontitis between the NF1 patients and NF1- controls [20, 27–29]. The statistical software used was GraphPad Prism, version 5 (GraphPad Software, Inc).

### Ingenuity pathway analysis

Canonical Pathway Analysis was conducted by comparing differentially expressed (DE) ribonucleic acid (RNA)-sequencing (RNA-seq) data registered in Gene Expression Omnibus of the National Centre for Biotechnology Information. We compared RNA-sequencing data from whole-transcriptome expression profiling using z-score and *P*-value visualization to identify underlying mechanisms among peripheral blood from patients with NF1, periodontium tissue isolated from patients with periodontitis, and parotid gland tissue from patients with sicca syndrome, compared with healthy controls. The DE RNA-seq was derived by normalizing the RNA expression level of patient samples with that of healthy controls. RNA-seq data with values of − log_10_(P) larger than 1.3 were considered significant, and positive z-scores indicated up-regulation while negative scores indicated down-regulation. The canonical pathway analysis was conducted with Ingenuity Systems Pathway Analysis (IPA) software (QIAGEN, Hilden, Germany). Additionally, the network between NF1 and periodontitis or sicca syndrome/Sjogren’s syndrome was constructed through Path Explorer and Molecular Activation Prediction (MAP) within IPA, based on reported gene–gene interactions in Gene Expression Omnibus.

## Results

### Demographics of NF1 patients and NF1- controls

Out of the 11 NF1 patients, 5 were females and 6 were males. In these 11 individuals, 4 aged below 21 years, 4 aged between 21 and 40, and 3 aged over 40 years. Controls were matched for age (+/− 3 years ), gender, residing region, socio-economic status, personal and deleterious habits, and medications, including calcium channel blockers and caffeine. The case-to-control ratio was 1-to-2.6 (Appendix Table 2). The mean ages of NF1 patients and NF1- controls were 35.18 and 38. 96, respectively. After adjusting for the above-mentioned matching criteria, there was no significant difference between NF1 patients and NF1- controls (Appendix Table 2).

**Table 1:** Presentation of oral complications among NF1 patients and NF1- controls

Index	Age subgroups	Mean of NF1- controls	Mean of NF1 + cases	Mean difference (95% CI) = values of NF1 + —that of NF1-	*P* value
Salivary flow rate	< 20	0.340	0.335	− 0.005 (− 0.019–0.0092)	0.45
Salivary flow rate	21–40	0.321	0.293	− 0.029 (− 0.047– − 0.011)	0.005
Salivary flow rate	> 40	0.292	0.173	− 0.12 (− 0.15– − 0.09)	< 0.0001
Amylase activity	< 20	0.387	0.374	− 0.013 (− 0.03–0.0083)	0.15
Amylase activity	21–40	0.377	0.323	− 0.053 (− 0.075– − 0.033)	0.0008
Amylase activity	> 40	0.360	0.292	− 0.068 (− 0.089– − 0.047)	< 0.0001
Salivary pH value	< 20	7.04	7.08	0.032 (− 0.068–0.13)	0.49
Salivary pH value	21–40	7.10	7.80	0.70 (0.46–0.94)	< 0.0001
Salivary pH value	> 40	7.17	8.27	1.10 (0.911.27)	< 0.0001
PI	< 20	0.49	0.66	0.16 (− 0.016–0.35)	0.07
PI	21–40	1.46	3.70	2.25 (0.69–3.80)	0. 009
PI	> 40	4.80	6.53	1.73 (0.16–3.31)	0.03
DMFT score	< 20	1.86	1.25	− 0.61 (− 2.80–1.59)	0.55
DMFT score	21–40	4.62	9.5	4.88 (0.33–9.41)	0.04
DMFT score	> 40	7.71	22.67	14.95 (12.44–17.47)	< 0.0001

### Periodontal findings and dental caries in NF1 patients and NF1- controls

**Fig. 1:**
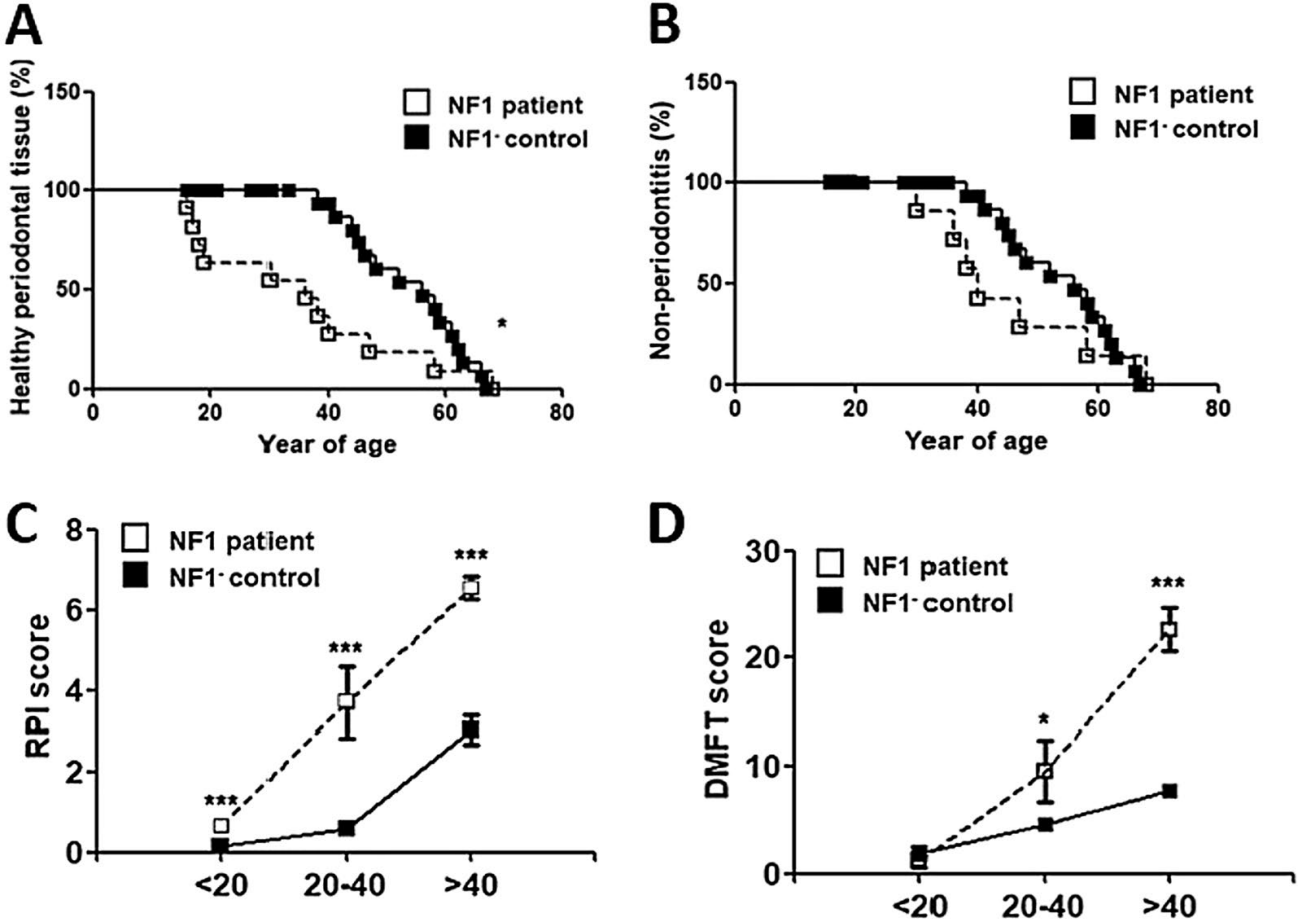
Periodontal diseases and DMFT among NF1 patients and NF1- controls. **A** Prevalence of gingivitis diagnosed clinically among NF1 patients and NF1- controls (OR = 1.56, 95% CI = 1.10–2.02, *P* = 0.0002). **B** Prevalence of periodontitis among NF1 patients and NF1- controls (OR = 1.40, 95% CI = 1.06–1.74, *P* = 0.04). **C** Periodontal indices among NF1 patients and NF1- controls. Y axis represents PIs. X axis represents age of participants. **D** DMFT scores among NF1 patients and NF1- controls. Y axis represented DMFT scores. X axis represented age of participants

The prevalence of gingivitis (OR = 1.56, 95%CI = 1.10–2.02, *P* = 0.0002) (Fig. 1A) and periodontitis (OR = 1.40, 95%CI = 1.06–1.73, *P* = 0.04) (Fig. 1B) were significantly higher in NF1 patients compared to that of NF1- controls. Subgroup analyses based on age stratification suggested that the PIs and DMFT scores were age-dependent, which were both significantly higher among NF1 patients aged over 20 (Fig. 1C for PI scores and Fig. 1D for DMFT).

Specifically, the PIs were 3.7 for NF1 patients versus 1.45 for NF1- controls aged between 20–40 (mean difference = 2.24, 95%CI = 0.70–3.80, *P* = 0.009), and 6.53 for NF1 patients versus 4.8 for controls aged over 41 (mean difference = 1.73, 95% CI = 0.16–3.30, *P* = 0.03). In contrast, the PIs were not significantly different among NF1 patients below 20 years old (0.65 versus 0.49, mean difference = 0.16, 95%CI =  − 0.01–0.34, *P* = 0.069) (Table 1).

Likewise, the DMFT scores were 9.5 for NF1 patients versus 4.63 for NF1- controls aged between 20 and 40 (mean difference = 4.87, 95% CI = 0.33–9.41, *P* = 0.03), and 22.67 for NF1 patients versus 7.71 for NF1- patients aged over 41 (mean difference = 14.95, 95%CI = 12.44–17.47, *P* < 0.0001). In contrast, the DMFT scores were not significantly different among NF1 patients and NF1- participants below 20 years old (1.25 versus 1.85, mean difference =  − 0.61, 95% CI =  − 2.80–1.59, *P* = 0.55) (Table 1).

### Salivary changes in NF1 patients and NF1- controls

**Fig. 2:**
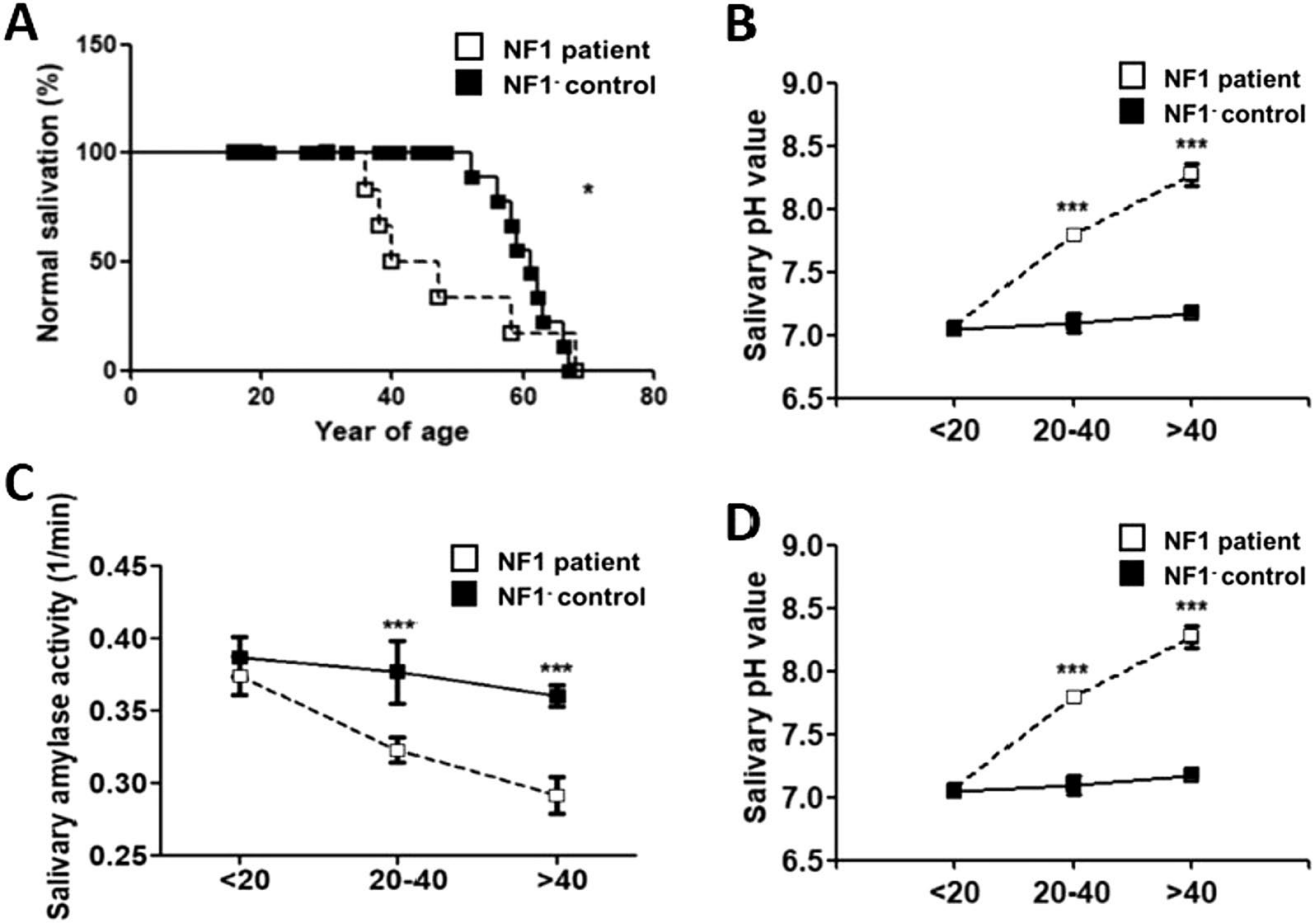
Comparison of salivary changes among NF1 patients and NF1-controls. **A** Prevalence of decreased salivary flow rate among NF1 patients and NF1- controls (OR = 1.40, 95% CI = 1.05–1.76, *P* = 0.005). **B** Whole unstimulated salivary flow rates among NF1 patients and NF1- controls. Y axis represented flow rates (ml/min). X axis represented age of participants. **C** Salivary amylase activity among NF1 patients and NF1- controls. Y axis represented amylase activity (1/min). X axis represented age of participants. **D** Salivary pH values among NF1 patients and NF1- controls. Y axis represented pH values. X axis represented age of participants

Significantly decreased salivary flow rates in NF1 patients (OR = 1.40, 95%CI = 1.05–1.76, P = 0.005)  (Fig. 2A) were noted. Overall, the salivary changes in NF1 patients were age-dependent. Subgroup analyses based on age stratification suggested that the whole unstimulated salivary flow rates (ml/min) and the salivary amylase activity (1/min) were age-dependent, which were both significantly low among NF1 + patients, aged over 20 (Fig. 2B for salivary flow rates and Fig. 2C for salivary amylase activity). Moreover, the salivary pH values were significantly high among NF1 patients aged over 20 (Fig. 2D).

For instance, the flow rates were 0.29 for NF1 patients versus 0.32 for controls aged between 20 and 40 (mean difference =  − 0.029, 95%CI = 0.047– − 0.011, *P* = 0.005), and 0.17 for NF1 patients versus 0.29 for controls aged over 41 (mean difference =  − 0.12, 95% CI =  − 0.14–0.088, *P* < 0.0001). On the contrary, the flow rates were not significantly different among NF1 patients below 20 years old (0.34 versus 0.34, mean difference =  − 0.005, 95% CI = 0.019–0.0091, *P* = 0.44) (Table 1).

Likewise, amylase activity, which was defined as the reciprocal number of the time for amylase to function, was 0.32 (min^−1^) for NF1 patients versus 0.38 (min^−1^) for NF1- controls aged between 20 and 40 (mean difference =  − 0.054, 95% CI =  − 0.075 to 0.033, *P* = 0.0008), and 0.29 (min^−1^) for NF1 patients versus 0.36 (min^−1^) for NF1- controls aged over 41 (mean difference =  − 0.068, 95% CI =  − 0.089 to − 0.046, *P* < 0.0001). But the times were not significantly different among NF1 + individuals below 20 years old (0.37 versus 0.39, mean difference =  − 0.013, 95% CI =  − 0.034–0.0082, *P* = 0.15) (Table 1).

Finally, the pH values were 7.8 for NF1 patients versus 7.1 for NF1- controls aged between 20 and 40 (mean difference = 0.7, 95%CI = 0.46–0.94, *P* < 0.0001), and 8.27 for NF1 patients versus 7.17 for controls aged over 41 (mean difference = 1.10, 95%CI = 0.91–1.27, *P* < 0.0001). In contrast, the pH values were not significantly different among NF1 patients below 20 years old (7.07 versus 7.04, mean difference = 0.032, 95%CI =  − 0.068–0.13, *P* = 0.48) (Table 1 & Fig. 2).

### Genetic and cellular mechanisms underlying NF1, salivary gland dysfunction, and periodontal destruction

**Fig. 3:**
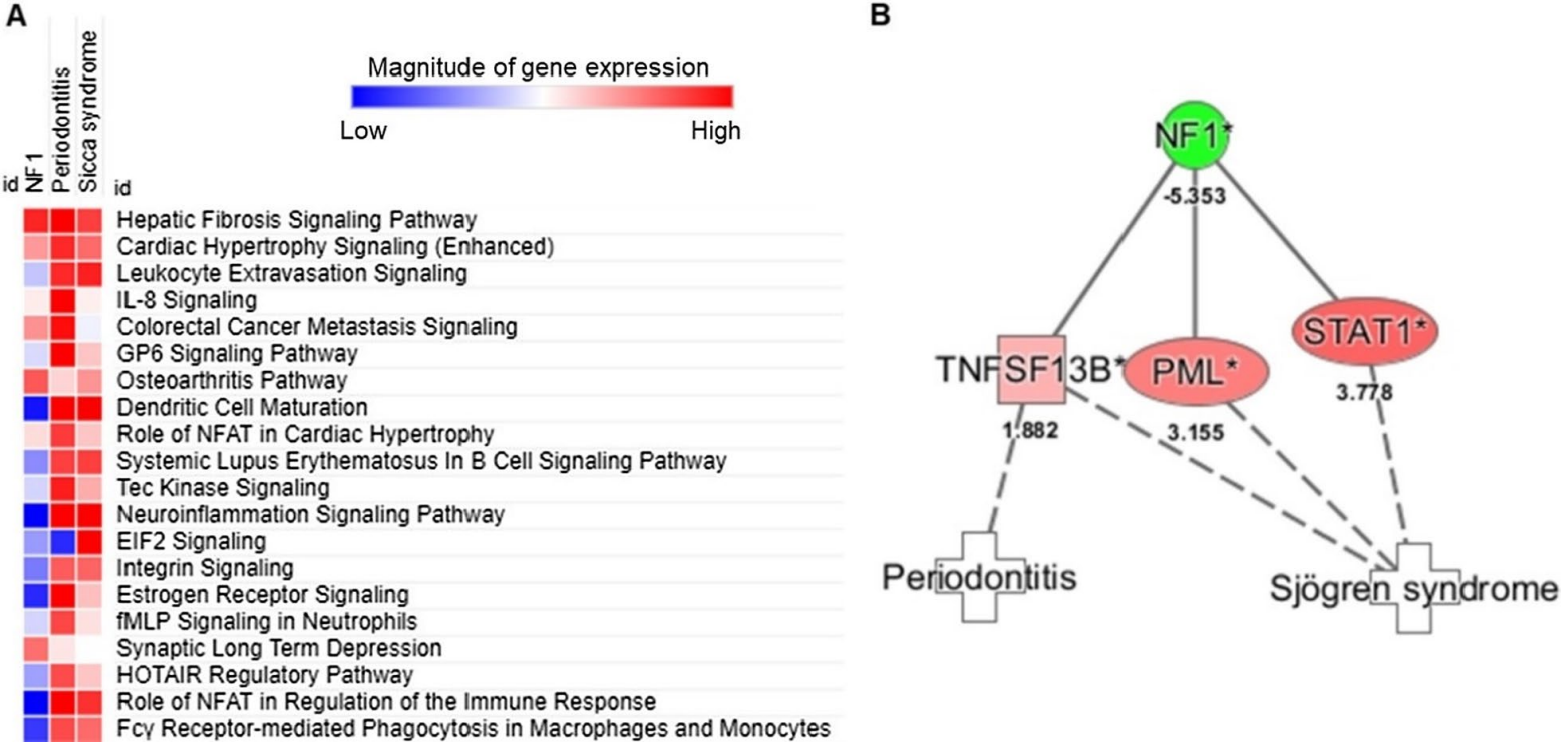
NF1-aasociated pathways and genes in periodontitis or sicca syndrome identified by IPA. **A** Canonical pathway analysis was performed with RNA-seq data of dermal neurofibroma-derived Schwann cells from patients NF1 (n = 3, GSE14038), gingival tissues from patients with periodontitis (n = 3, GSE23586), as well as parotid glands from patients with sicca syndrome (n = 3, GSE66795). Top 20 NF1-associated canonical pathways for periodontal destruction and salivary gland dysfunction were listed. **B** IPA analyses showed putative network generated using published (solid lines) and predicted (dashed lines) between NF1 and periodontitis or sicca syndrome

IPA analyses indicated that the possible mechanisms underlying the correlation between NF1 with cutaneous neurofibromas and oral complications including (1) periodontal destruction, and (2) salivary gland dysfunction, involved multiple chronic inflammatory pathways (Fig. 3A). As for the top 20 canonical pathways mutually expressed in the pathogenesis of NF1, periodontitis, and sicca syndrome, which were ordered based on the sum of z scores, fibrosis signaling pathway was highly expressed in the three disease groups when compared to healthy donors, with Z scores being 3.70 for NF1, 4.54 for periodontitis, and 3.5 for sicca syndrome, and significant − log_10_(P) values being 11.28 for NF1, 10.66 for periodontitis, and 3.30 for sicca syndrome, respectively.

**Fig. 4:**
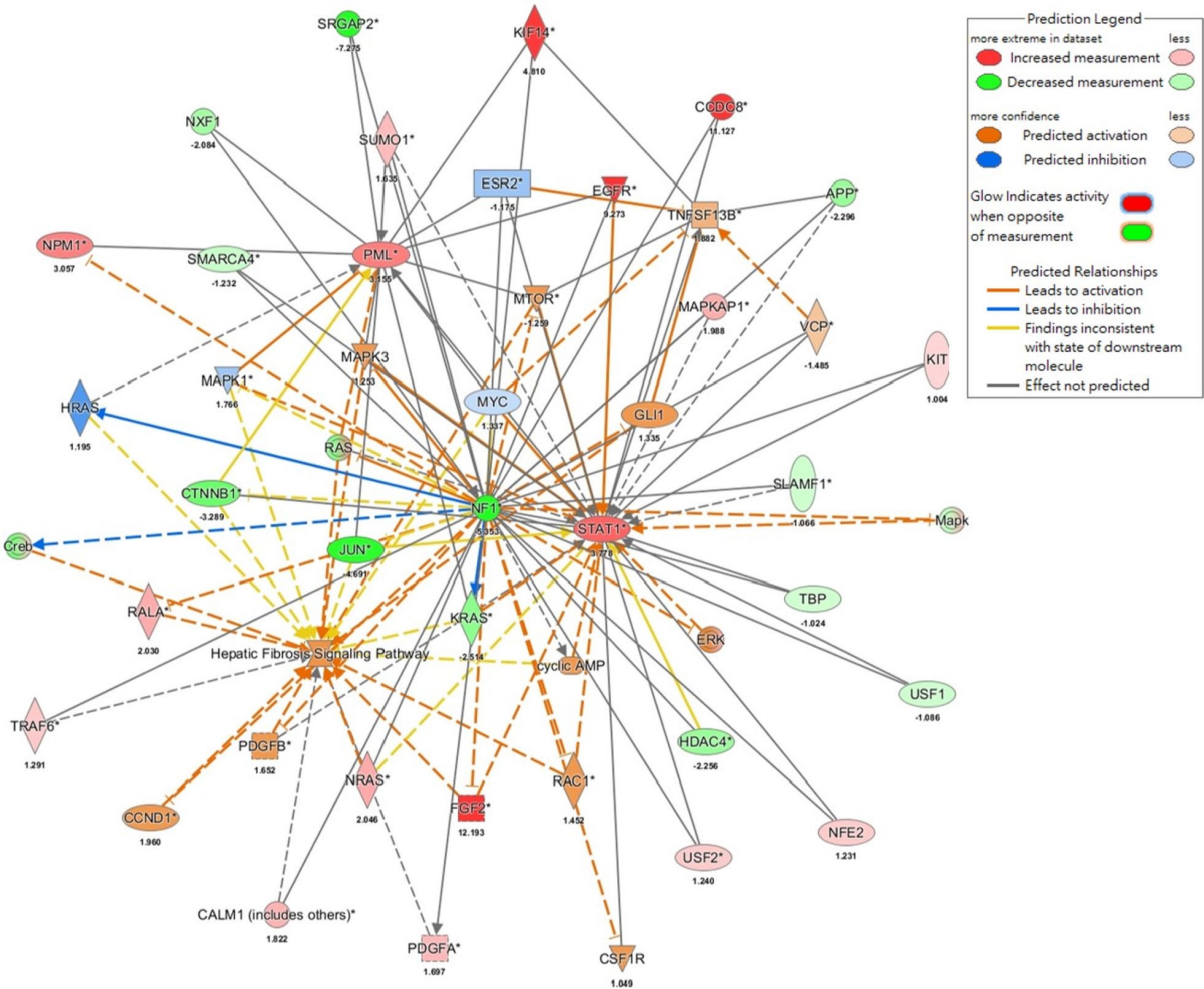
Downregulation of NF1 gene induced fibrosis through upregulating the expression levels of TNFSF13B-, PML, and STAT1-dependent pathways in NF1 patients. The network of NF1-associated fibrosis was built in IPA. The mechanisms through which activation of TNFSF13B, PML and STAT1 led to canonical fibrosis pathway were derived using Molecular Activation Prediction

Path Explorer performed using IPA suggested that the downregulation of NF1 gene in NF1 patients may induce periodontitis via upregulation of TNF Superfamily Member 13b (TNFSF13B) while linking to sicca syndrome or Sjögren's syndrome with upregulated TNFSF13B, promyelocytic leukaemia (PML) and signal transducer and activator of transcription 1 (STAT1) (Fig. 3B). Moreover, MAP within IPA demonstrated that the lack of NF1 expression resulted in the upregulation of fibrosis pathway through TNFSF13B-, PML-, or STAT1-involved pathways (Fig. 4). These results demonstrated the upregulated pathways and genes underlying the association between NF1 and the observed oral complications in this study, including periodontal destruction and sicca-like symptoms.

## Discussion

In this case–control study, NF1 patients were associated with periodontal disease, decreased salivary flow rates, compromised amylase activities, and high prevalence of caries, all in an age-dependent manner. Significantly high PI scores and DMFT scores, low unstimulated salivary flow rates and amylase activities, and high salivary pH values, were observed among NF1 patients aged over 20 years old. For those aged below 20 years, the above-mentioned measurables were not significantly different between NF1 patients and NF1- controls. Moreover, the difference in the means was the highest for those aged over 40, followed by those aged between 20 and 40, finally those aged below 20. With RNA-seq data from peripheral blood in NF1 patients, gingival tissues from patients in periodontitis, and parotid glands in patients with sicca syndrome, IPA analysis predicted that several chronic inflammatory pathways were involved for the association. These pathways included the fibrosis pathway, which may initiate periodontitis and sicca-like symptoms through the upregulation of TNFSF13B, PML and STAT1.

Although a case of gingival neurofibroma in the attached gingiva has been reported [30, 31], so far there is a lack of evidence regarding whether NF1 patients may present periodontal disease without tumors directly involving the oral cavity. In our study, as all NF1 patients did not have neurofibromas in the maxilla or mandible, our findings for the first time demonstrated that NF1 patients were at a high risk of periodontitis. As periodontitis is an inflammatory disease triggered by dysbiosis in the oral cavity [32, 33], our findings on periodontitis in patients with NF1 were in accordance with previous studies pinpointing that NF1 patients presented with low-grade chronic inflammation [34]. On the other hand, our findings on salivary gland dysfunction and dental caries were in phase with a previous case–control study in which the prevalence of hyposalivation in NF1 patients was four-fold higher than their respective NF1- controls [13].

Apart from the above-mentioned theory that low-grade chronic inflammation in NF1 patients may initiate or exacerbate periodontal disease [13], a previous cross-sectional study conducted on 150 individuals demonstrated that the saliva was more alkaline among individuals with periodontitis, which was similar to our study [25] in that high salivary pH values were noted in the enrolled NF1 patients. Importantly,  alkaline changes to pH values of the saliva can lead to a decrease in the activity of various salivary anti-microbial proteins and enzymes, as evidenced by salivary amylase activities in starch-iodine-saliva experiments**.** The basic environment of saliva can also increase the proteolytic activity of the organism, which promotes the deposition of calcium phosphate, thereby placing NF1 patients at great risk of dental caries and periodontal destruction as a feedback loop [25].

Other factors contributing to periodontitis and caries in NF1 patients include fused tooth, misregulation of amelogenesis leading to enamel hypoplasia, microdontia [14], low levels of serum 25-hydroxy vitamin D3 and increased osteoclastic activity [35]. Specifically, low levels of serum 25-hydroxy vitamin D3 among NF1 patients may result from increased pigmentation, cutaneous neurofibromas-associated impaired synthesis or accelerated catabolism, with its severity increasing with age, making NF1 patients susceptible to periodontitis in an age-dependent pattern [36]. Moreover, histological evidence revealed that the infiltration of mast cells through stem cell factor (SCF) can drive STAT1 activation, creating an inflammatory microenvironment in neurofibroma that is considered a clinicopathological hallmark [37, 38]; on the other hand, the increase of mast cells, also known as mastocytosis, is as well critically involved in the pathogenesis of periodontitis through secreting inflammatory cytokines and participating in periodontal tissue destruction [39]. As such, mast cells may play an essential pathological role in connecting neurofibromatosis and periodontitis.

Several studies proposed mechanisms that can explain salivary gland dysfunction in NF1 patients or models. For instance, animal studies suggested that the NF1 gene played an important role in the organogenesis of salivary glands [12]. Likewise, a study based on salivary gland tissues from NF1 patients pointed out that the neurofibromin gene was expressed in major and minor salivary acinar, ductal epithelium irrespective of age and sex, which indicated the importance of the NF1 gene in the normal function of the salivary gland [40]. Overall, mechanisms that may contribute to hyposalivation in NF1 patients included defective organogenesis of the salivary gland [41, 42], parasympathetic ganglion supplying the gland [42], and neurovascular bundles determining the quality and quantity of saliva that enter the salivary glands [41].

Moreover, mutations of the NF1 gene were reported to bring about complications that resulted from collagen loss or inability to produce collagen fibers, with which the underlying fibrosis pathways may account for pulmonary complications of NF1, such as interstitial lung disease and pre-capillary pulmonary hypertension [43–45], lack of skin elasticity [46], and the susceptibility to osteoporosis and osteomalacia [47] Thus, similar to how the fibrosis of alveolar epithelium and connective tissue of lungs result in pulmonary complications of NF1 [43–45], the effect of mutations of the NF1 gene on the observed gingival tissue loss/ periodontal destruction and sicca-like symptoms can also be underlain by an inadequate collagen amount in the connective tissue of both the periodontium and salivary glands in patients with NF1 [48]. As salivary glands function through squeezing the saliva out of these exocrine glands [49] that are full of type I collagen [50, 51], inadequate collagen production or defective collagen structure [46–48] in the context of NF1 may lead to adverse salivary changes, such as abnormal salivary flow rates and salivary pH values that were noted in the present study.

The major strengths of this study included matching between NF1 patients and NF1- controls, with which the potential effect of confounding factors such as age, gender [52], region, socioeconomic status, medical history, personal and deleterious habits [53] on periodontal health and salivary flow was minimized. However, limitations of this study included the inability to provide robust causal inference in case–control studies of relatively small sample sizes. Moreover, due to the high mutation spectrum of NF1-associated genes, for which genotype–phenotype correlations had been widely investigated for various phenotypic expressions of NF1 [54–58], the lack of genetic sequencing data that can provide information on NF1-mutated subtype limited the ultimate scope to delineate specific mutated regions that were associated with the observed intraoral complications in this study. Future studies incorporating radiographics and genetics are warranted to provide more details about the presenting orofacial complications of NF1, and to provoke patient awareness through communication [59] and education [60].

In conclusion, this study demonstrated age-dependent periodontal destruction and functional salivary changes correlating with carious lesions in NF1 patients, which were evidenced by intraoral examinations and functional salivary tests. These oral complications, instead of resulting from direct compression by neurofibromas, may develop following systemically upregulated chronic inflammatory pathways.

## Data Availability

The data used in this study are available upon reasonable requests, and all consent forms will be provided with request.

## Appendix

See Table 2.

**Table 2:** Baseline characteristics for NF1 + and NF1- controls

Characteristics	NF1- controls	NF1 + cases	Case/ control (a) or *P*-value (b)
Number	29	11	1: 2.6 (a)
Age distribution			
< 21	7	4	1:1.7 (a)
21–40	8	4	1:2 (a)
> 40	14	3	1: 4.6 (a)
Mean age (years)	38.96	35.18	–
Sex (female/male*100%)	48.2%	45.4%	–
Tobacco use	6	2	0.6 (b)
Alcohol	5	2	0.6 (b)
Medication history of xerostomia-associated drugs	6	2	0.6 (b)
